# *AUSPEX*: a graphical tool for X-ray diffraction data analysis

**DOI:** 10.1107/S205979831700969X

**Published:** 2017-08-08

**Authors:** Andrea Thorn, James Parkhurst, Paul Emsley, Robert A. Nicholls, Melanie Vollmar, Gwyndaf Evans, Garib N. Murshudov

**Affiliations:** a Hamburg Centre for Ultrafast Imaging, Universität Hamburg, Luruper Chaussee 149, 22761 Hamburg, Germany; b Diamond Light Source, Harwell Science and Innovation Campus, Didcot OX11 0DE, England; c MRC Laboratory of Molecular Biology, Francis Crick Avenue, Cambridge Biomedical Campus, Cambridge CB2 0QH, England

**Keywords:** ice rings, macromolecular crystallography, data processing, data analysis, X-ray diffraction, data quality, *AUSPEX*

## Abstract

In this paper, *AUSPEX*, a new software tool for experimental X-ray data analysis, is presented. Exploring the behaviour of diffraction intensities and the associated estimated uncertainties facilitates the discovery of underlying problems and can help users to improve their data acquisition and processing in order to obtain better structural models. The program enables users to inspect the distribution of observed intensities (or amplitudes) against resolution as well as the associated estimated uncertainties (sigmas). It is demonstrated how *AUSPEX* can be used to visually and automatically detect ice-ring artefacts in integrated X-ray diffraction data. Such artefacts can hamper structure determination, but may be difficult to identify from the raw diffraction images produced by modern pixel detectors. The analysis suggests that a significant portion of the data sets deposited in the PDB contain ice-ring artefacts. Furthermore, it is demonstrated how other problems in experimental X-ray data caused, for example, by scaling and data-conversion procedures can be detected by *AUSPEX*.

## Abstract 2

*AUSPEX* is a new software tool for the statistical analysis of single-crystal X-ray diffraction data. It can be used to identify problems in the data resulting from the experiment itself, image processing, data scaling or conversion.

## 1. Introduction   

### 1.1. Diagnostic tools for the processing and interpretation of X-ray diffraction data   

Diagnostic tools are important in all stages of data modelling and data analysis as they provide information about the quality of the data and the model, as well as their relationship to one another. In crystal structure determination, diffraction data are interpreted using an atomic model of the unit-cell content of the crystal and its lattice symmetry. Consequently, diagnostic tools are important to derive reliable atomic models from diffraction data. In particular, graphical tools provide a fast and convenient representation of data and can reveal problems which might otherwise go unnoticed. As well as being valuable for users, they are also important to methods developers during the design and improvement of algorithms.

However, whilst some of the data-processing software for macromolecular crystallography produces plots of averaged reflection intensities against resolution, individual data points for each reflection are not usually shown. Producing the latter is well within the capability of modern computer systems and, as we will demonstrate in this paper, it can reveal artefacts in the data, such as the presence of so-called ice rings, which may otherwise be hidden by averaging.

### 1.2. Ice rings   

Ice rings are Debye–Scherrer rings observed at specific resolutions as a result of X-ray diffraction from a multitude of arbitrarily oriented, typically hexagonal or cubic, ice crystals (Garman & Owen, 2006 ▸). Single-crystal X-ray diffraction experiments are routinely carried out at cryogenic temperatures and almost all crystalline samples of biological macromolecules are grown from aqueous media. As a result, ice rings are a common occurrence in the diffraction from such samples.

**Figure 1:**
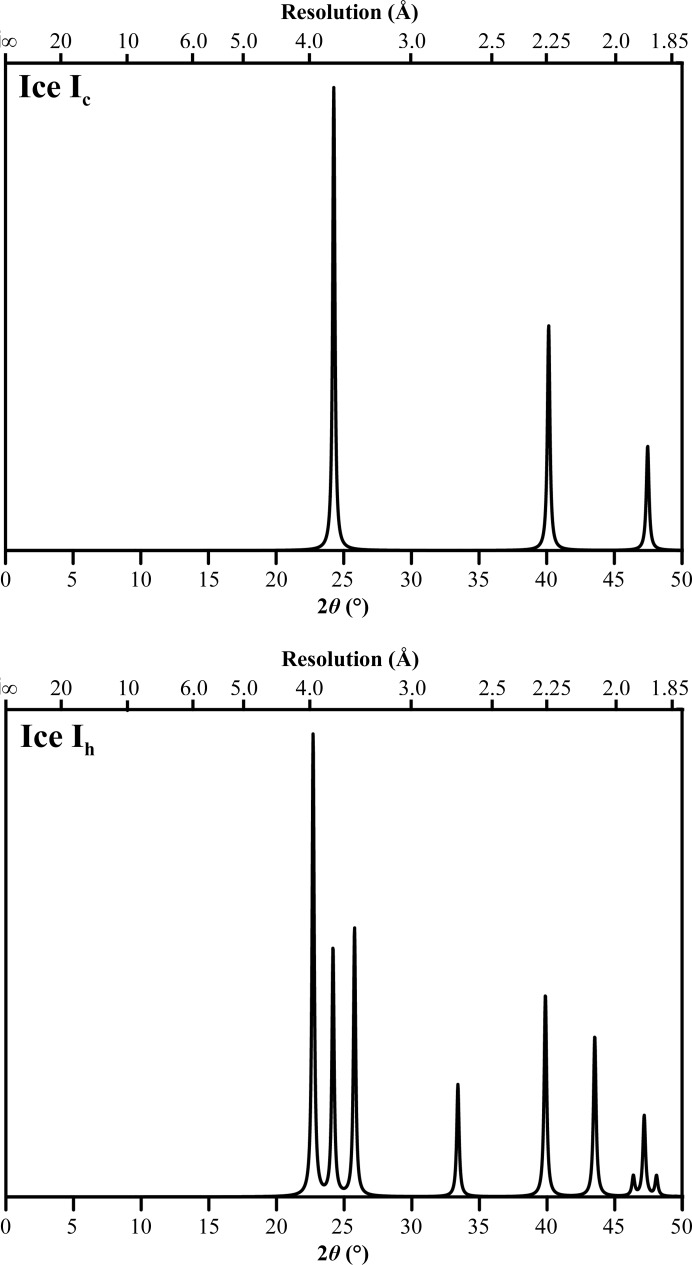
Theoretical powder diffraction peak profiles for hexagonal ice (*I*_h_) and cubic ice (*I*_c_) at the Cu *K*α wavelength calculated with *XPREP* (v.2015/1; Bruker-AXS). For the observed powder diffraction patterns, see Fuentes-Landete *et al.* (2015 ▸).

Cubic (I_c_) and hexagonal (I_h_) ice exhibit similar interatomic distances and have similar volumetric mass density, and both exist at ambient pressure. Hexagonal ice is the more common of the two forms. Cubic ice can be seen as a metastable form of hexagonal ice, as it occurs only as nanocrystals and with hexagonal stacking (Fuentes-Landete *et al.*, 2015 ▸). The theoretical powder diffraction peak profiles of I_h_ and I_c_ ice are shown in Fig. 1 ▸.

Ice rings can cause problems in data processing and modelling. Maximum-likelihood methods, as implemented in, for example, *Phaser* (McCoy *et al.*, 2007 ▸), *SHARP* (de La Fortelle & Bricogne, 1997 ▸), *REFMAC*5 (Murshudov *et al.*, 1996 ▸) and S-SAD (Hendrickson & Teeter, 1981 ▸), are widely used for phasing and refinement in macromolecular crystallography. However, they are particularly sensitive to departures from the assumptions made about the statistical distribution of data, which can be caused for example by outliers, unmodelled observations or incorrect error estimates (Waterman & Evans, 2010 ▸). Ice rings can result in a systematic bias to the estimated reflection intensities from integration programs; this reduces the information transferred from the data to the atomic model, and may in extreme cases even prevent structure solution.

Three strategies are currently available to address the problem during data processing.

**Figure 2:**
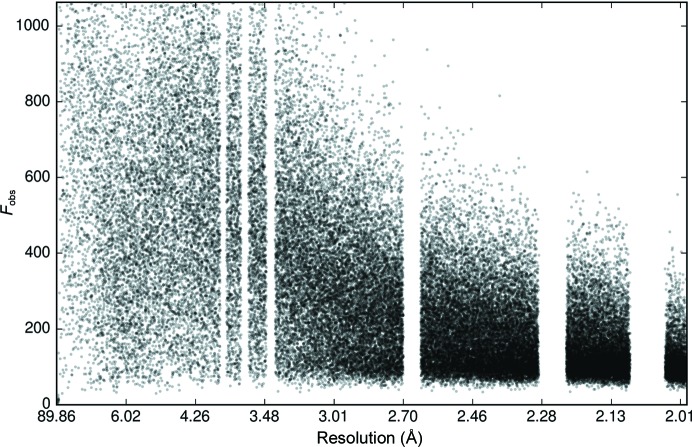
*AUSPEX* plot of *F*_obs_ *versus* resolution for PDB entry 4puc, the structure of a SusD homologue determined by the Joint Centre for Structural Genomics; no integration program was given for these deposited data, but scaling was performed with *XSCALE* (Kabsch, 2010 ▸, 2012 ▸). Masking out of the ice rings in integration led to a significant omission of data: the overall completeness is 78.1%.

**Figure 3:**
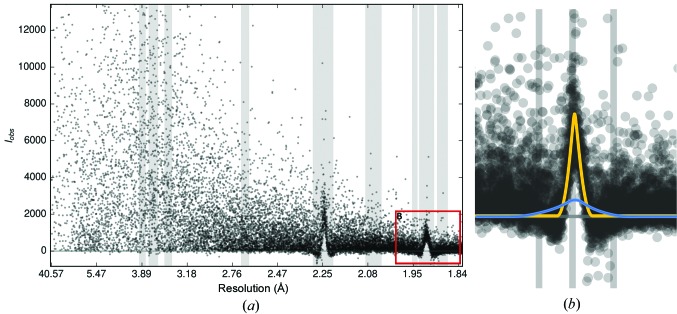
(*a*) *AUSPEX* plot of *I*_obs_ *versus* resolution for PDB entry 4epz. This data set was processed with *DIALS* and scaled with *AIMLESS* (Evans & Mushudov, 2013 ▸). For hexagonal ice there should be nine rings visible at this resolution (see Fig. 1 ▸), while for cubic ice there should be three (at 3.67, 2.25 and 1.92 Å). The resolution ranges corresponding to potential ice rings are marked using grey bars (whether hexagonal or cubic ice). Two ice rings are clearly visible at high resolution, while the other (at least one more must be present even for cubic ice at 3.67 Å) was successfully modelled in integration. Hence, when identifying ice rings in integrated data, the presence of all ice rings in question is not a reliable criterion. (*b*) Background overestimation and underestimation: this enlarged view of the ice ring at 1.918 Å shows the effects of insufficient background correction. The blue line shows the likely background as assumed by the integration program (*DIALS*). The yellow line shows the likely background caused by ice. The discrepancy causes an underestimation of *I*_obs_ values to the left and right of the ice ring, resulting in large negative intensity values, and an overestimation of *I*_obs_ in the ice ring, resulting in a peak in *I*_obs_ values.

(i) Resolution ranges contaminated by ice diffraction can be omitted (see Fig. 2 ▸). This requires manual intervention to determine the presence of an ice ring at a certain resolution (Kabsch, 2010 ▸) and may reduce completeness significantly and systematically, likely distorting the resulting electron-density maps.
(ii) All integration programs estimate the background under a reflection (Leslie, 1999 ▸; Kabsch, 2010 ▸; Parkhurst *et al.*, 2016 ▸); if the ice ring is broad enough (*i.e.* it covers the entire integration box for a reflection) and the background is modelled by a plane, the background estimation may account for an ice ring. However, if an ice ring is narrow or irregular then current background models can fail to account for this diffraction (see Fig. 3 ▸).
(iii) Images can be pre-processed (Chapman & Somasundaram, 2010 ▸) to remove the background created by ice rings. This might not be ideal as it may fail to account for detector tilt or directionally dependent variation in the background.

Because none of these strategies are universally applicable and result in a complete data set without any ice-ring contamination, the optimization of cryoconditions (the conditions under which a crystal is cooled to the desired temperature) is an important step in macromolecular crystallography (Mitchell & Garman, 1994 ▸). Suitable conditions show diffraction of the macromolecular specimen without ice diffraction: ice rings are typically detected by the inspection of X-ray diffraction images, often during cryocondition optimization (Mitchell & Garman, 1994 ▸) or during data collection. In addition to the inspection of detector images, as early as 1996 McFerrin and Snell proposed the use of resolution-averaged intensity, then called ‘powder integrated intensity’, as an indicator of the presence of ice rings.

**Figure 4:**
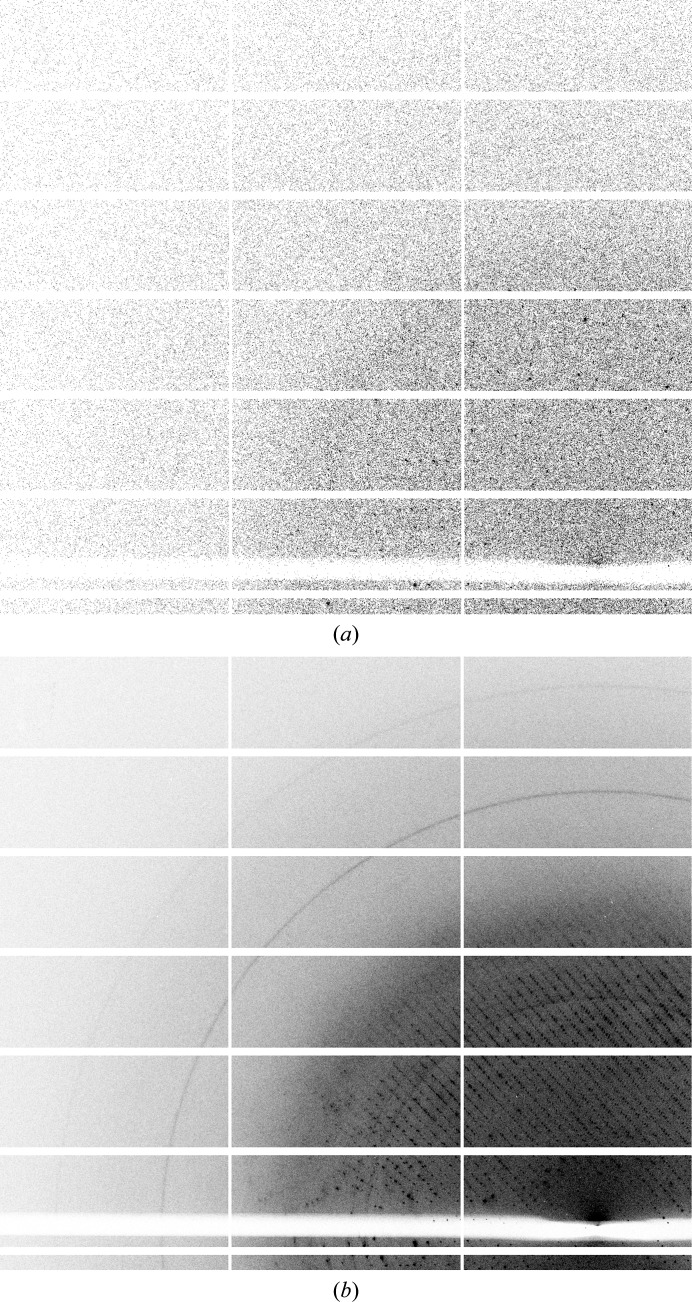
Ice-ring data on a pixel-detector image. Ice rings are not always visible on the detector, and this problem is aggravated by the usage of fast-readout pixel-array detectors, where the measurement rotation range is typically ‘sliced’ very finely. (*a*) Single image with 0.15° rotation. (*b*) 100 images from the same data set summed: ice rings are visible.

The need for alternative means of ice-ring detection has recently been emphasized by the proliferation of pixel detectors with millisecond readout times. This readout speed and the low noise in images from such detectors poses an advantage, and images from such detectors usually cover a smaller angular increment (‘fine slicing’) than images collected using earlier detector technologies, leading to shorter exposure times. Consequently, ice rings (and other background-related problems) are hard to identify by visual inspection of single images alone. They become more evident if images are summed together, for example with *DIALS* (Waterman *et al.*, 2013 ▸), to produce a ‘stacked image’, as shown in Fig. 4 ▸.

In the presence of an ice ring, the calculated structure-factor amplitudes *F*_calc_ and observed structure-factor amplitudes *F*_obs_ (derived from peak integration of the X-ray image) diverge noticeably. Therefore, ice rings are also visible as outliers in plots of the crystallographic *R* value or similar indicators against resolution.

However, after data integration and scaling, and before structure solution, ice rings are more difficult to identify because two-dimensional information from the diffraction image has been reduced by data integration and a structural model is not yet available for comparison. After data reduction, only two currently available programs give an indication of ice-ring contamination: *phenix.xtriage* (Zwart *et al.*, 2005 ▸) and *CTRUNCATE* (Winn *et al.*, 2011 ▸).

In order to address the need for a more detailed analysis and representation of data at this stage, a new software tool, *AUSPEX* 1, is presented here. It can be used to detect the presence of ice rings and analyse X-ray diffraction intensities and their estimated standard uncertainties after integration and before a structural model is available.

### 1.3. Outline of this paper   

In this paper, we will first describe how a preliminary study on data from the Joint Centre for Structural Genomics (JCSG; Elsliger *et al.*, 2010 ▸) led to the development of *AUSPEX*, which we then used to evaluate 200 randomly selected structures from the PDB (Berman *et al.*, 2003 ▸). Subsequently, the automatic ice-ring detection is described and compared with other methods that are currently available. We then show examples of how *AUSPEX* can be used to identify other features in the data.

For the purposes of this article, *I*_obs_ and *F*_obs_ relate to the observed values of intensity and structure-factor amplitude, respectively, after data integration and scaling.

## 2. Preliminary study   

In a preliminary study, 156 integrated and scaled data sets from the JCSG measured using PILATUS detectors and deposited in the PDB between 2011 and 2015 were evaluated (test set A; see Supporting Information). The observed amplitudes *F*_obs_ were plotted against resolution. In these plots, ice rings were visibly identifiable in 15 of the 156 data sets (for an example, see Fig. 3 ▸). This indicates that the background estimation used in processing these data sets was insufficient to correct for the presence of ice diffraction. Two further data sets were found to have significant portions of data removed prior to processing owing to the presence of ice rings (see Fig. 2 ▸). It was clear that these plots held some diagnostic value, and as a result *AUSPEX* was developed.

## 3. Program description   

In the presence of an ice ring, negative intensities can result from incorrect estimation of the background (see Fig. 3 ▸). When intensities are subsequently converted to amplitudes, they must have values equal to or greater than zero (French & Wilson, 1978 ▸). Consequently, it was found that plots of observed intensities *I*_obs_ *versus* resolution, as shown in Fig. 3 ▸, are more useful than plots of observed amplitudes *F*_obs_ against resolution to identify ice rings in X-ray data sets.

**Figure 5:**
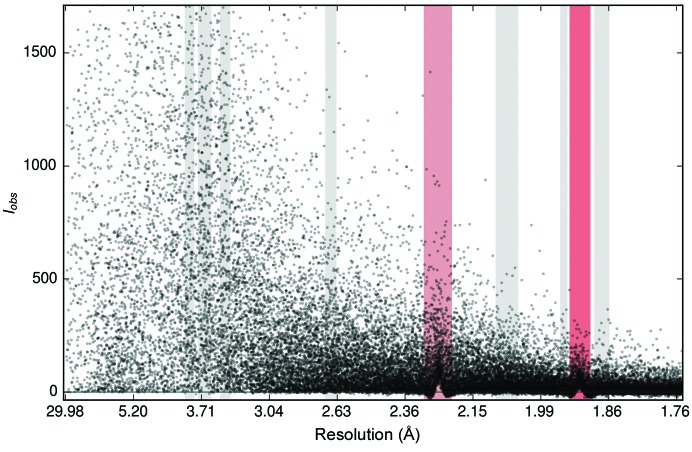
*AUSPEX* output for PDB entry 5kiv. Identified ice rings are flagged in red and typical ice-ring resolution ranges (see Table 1 ▸) are shown in grey. Intensity is plotted with the *y* axis limited. Note that an additional ice ring at 3.7 Å, which is barely visible, has not been flagged.

*AUSPEX* outputs graphs of *I*_obs_, *I*_obs_/σ(*I*_obs_) and σ(*I*_obs_) *versus* resolution. A typical output is shown in Fig. 5 ▸. In the absence of intensities, amplitudes (*F*_obs_) are used instead; if both are present then both sets of plots are generated, which can be useful for the evaluation of conversion to amplitudes. *AUSPEX* will then automatically detect the presence of ice rings as described in §[Sec sec5.1]5.1. *AUSPEX* is written in C++, with input and graphical output managed by a Python wrapper; the Clipper library (Cowtan, 2003 ▸) is used to perform crystallo­graphic calculations and *MATPLOTLIB* (Hunter, 2007 ▸) is used to generate plots. Currently, data sets must be input in MTZ format (McLaughlin & Terry, 1989 ▸).

## 4. *AUSPEX* usage examples   

In the following subsections, the use of *AUSPEX* to identify unusual data behaviour is demonstrated and the cause of the observed behaviour is investigated.

### 4.1. Ice-ring detection   

As shown in Fig. 3 ▸, if the background was poorly estimated during data integration then ice rings are visible as distinct peaks in the distribution of intensity against resolution, occurring at specific resolutions (see §[Sec sec1.2]1.2).

In order to quantify this problem in the PDB, we randomly selected 200 data sets from the PDB for which intensity values had been deposited (test set B; see Supporting Information). 145 had been measured on CCD detectors, 25 on pixel detectors and 16 on image plates. The rest had been measured using other detectors or no detector type was reported. There were no data sets in common with test set A. Plots of *I*_obs_ *versus* resolution for these data were generated using *AUSPEX* and were manually inspected for peak features similar to those shown in Figs. 2 ▸ and 3 ▸. Of the 200 data sets, which were collected between 1995 and 2015, 41 contained ice rings. (Three additional data sets contained ice rings and part of the data had been omitted in resolution bins; one data set had data omitted in resolution bins and no additional ice rings were visible in the rest of the data.) Inspection of other test sets from the PDB resulted in similar numbers (see, for example, §[Sec sec5.2]5.2). The high fraction of ice-ring-contaminated data sets clearly demonstrates the need for better background estimation and better diagnostic tools to alert users to the presence of ice rings.

### 4.2. Ice-ring shift   

In five of the 200 cases, ice rings were shifted in resolution from the typical ice-ring resolution ranges used in *AUSPEX* (see Table 1 ▸). The five cases in question (PDB entries 1nrj, 5ek4, 3mtl, 3wn2 and 5a30) were tested with the ‘Anomalous bond length’ feature in the *WHAT IF* online service (Vriend, 1990 ▸), which compares the bond distances in the model with standard values for protein and nucleic acid bond lengths. All five cases showed a significant systematic deviation according to *WHAT IF*. This, as well as the shift of the ice rings from their usual resolution ranges, may be caused by an error in the unit-cell dimensions, which is often the result of the use of an incorrect X-ray wavelength or detector distance during data processing (Thorn, 2011 ▸).

### 4.3. Effects of multiplicity on *I*_obs_/σ(*I*_obs_) at low resolution   

**Figure 6:**
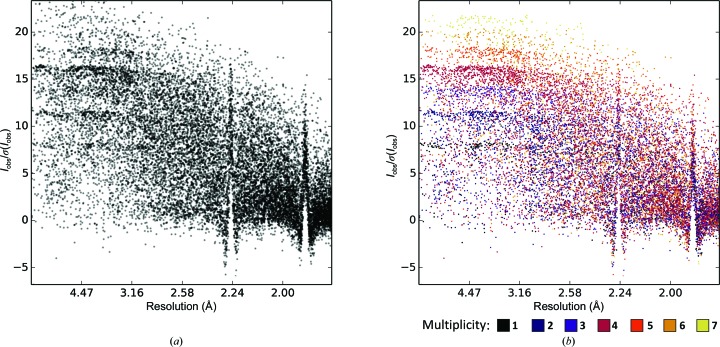
Plot of *I*_obs_/σ(*I*_obs_) *versus* resolution for PDB entry 4epz. (*a*) At high resolution ice rings are clearly visible, while at low resolution the values are clustered, forming a ‘ladder-like’ scatter plot. (*b*) The same plot coloured by multiplicity. The higher the multiplicity the larger *I*_obs_/σ(*I*_obs_) is, thus accounting for the behaviour. The value of a measurement is less uncertain the more often it has been made, as shown for example by Watkin & Cooper (2016 ▸). At low resolution, the main influence on these values is their multiplicity. In contrast, at high resolution weaker reflections are influenced by other factors.

Plots of *I*_obs_/σ(*I*_obs_) [and plots of *F*_obs_/σ(*F*_obs_)] *versus* resolution often show clustering around certain values at low resolution. When considering the associated multiplicity values (see Fig. 6 ▸), it was evident that the higher the multiplicity, the larger *I*_obs_/σ(*I*_obs_) is.

This is of course because when measurements are summed, and given that these measurements are independent of each other, their variances are summed as well. If the intensity *I* has been measured *N* times, denoted by *i*, then 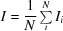
and 
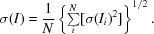
If all σ(*I*_*i*_) are equal, then 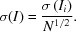



Hence, the ‘steps’ represent the discrete values of multiplicity in the data, *i.e.* the number of times a reflection, or its symmetry equivalent, has been measured. Increasing the number of individual measurements contributing to an average results in a lower σ(*I*_obs_) and therefore increases *I*_obs_/σ(*I*_obs_). However, at high resolution the accuracy of the data is usually limited by the crystal as well as the experimental setup (Diederichs, 2010 ▸), including the multiplicity, and hence the effect becomes less pronounced.

### 4.4. Conversion from intensities to amplitudes   

**Figure 7:**
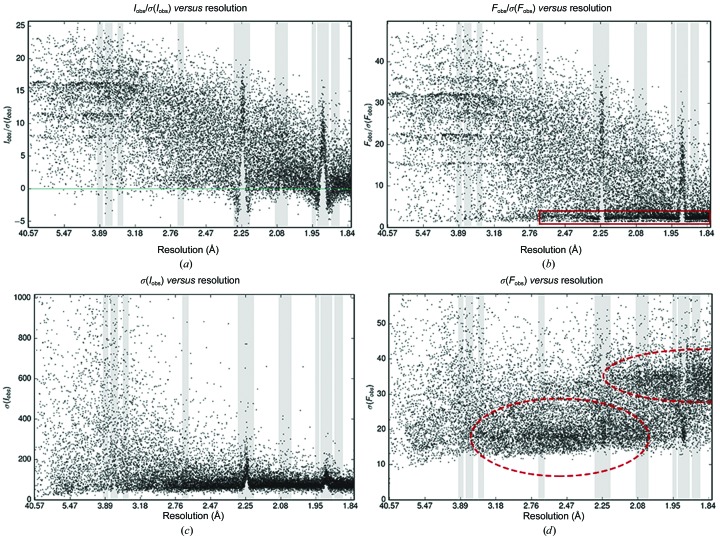
Effects of the conversion from intensities to amplitudes. (*a*) *I*_obs_/σ(*I*_obs_) *versus* resolution. (*b*) *F*_obs_/σ(*F*_obs_) does not contain any *F*_obs_/σ(*F*_obs_) equal to or smaller than zero. The centric reflections are visible as a thin line of values lower than the majority of the others (inside the red box). (*c*, *d*) While the ice rings have high σ(*I*_obs_), the σ(*F*_obs_) are dominated by the prior distribution, which is exponential or super-exponential, leading to smaller than average σ(*F*_obs_) for the ice-ring reflections. In addition, σ(*F*_obs_) values form two distinct clusters (red dashed ellipses), which is a typical effect of the conversion in *CTRUNCATE*.

*F*_obs_ is needed to calculate electron-density maps, and is also used as the observation against which many programs optimize structural models. Some exceptions are *Phaser*, where an intensity-based log likelihood target is used to avoid problems related to the conversion from *I*_obs_ to *F*_obs_ (Read & McCoy, 2016 ▸), *REFMAC*5 and *SHELXL* (Sheldrick, 2008 ▸), which refines against *I*_obs_. This also has the advantage of retaining all of the statistical properties, some of which (such as negative values) are lost in most conversion methods. Conversion from intensities *I*_obs_ to structure-factor amplitudes *F*_obs_ is usually performed using the French and Wilson algorithm (French & Wilson, 1978 ▸), which uses a Bayesian approach prior that forces negative *F*_obs_ values to be positive or zero-valued and Wilson-distributed. This prior may not be appropriate if the data are contaminated by ice rings (see §[Sec sec4.1]4.1) or if other systematic errors are present. The changes introduced by the conversion, as implemented for example in *CTRUNCATE*, can be illustrated by comparing *AUSPEX* plots of *I*_obs_/σ(*I*_obs_) with *F*_obs_/σ(*F*_obs_) (see Fig. 7 ▸).

## 5. Automatic ice-ring detection   

### 5.1. Implementation in *AUSPEX*   

The automatic ice-ring detection procedure considers the behaviour of the standardized mean 〈*I*_obs_〉/*s* in resolution bins, where 〈*I*_obs_〉 is the sample mean and *s* is the sample standard deviation of the intensities in a given bin. By default, equally spaced inverse-resolution bins of 0.001 Å^−1^ are used so as to achieve a reasonable compromise between binning fineness and noise.

Since data may contain various peculiarities, either inherent to the data or as a consequence of data processing, the observed average standardized mean 〈*I*_obs_〉/*s* may be systematically higher or lower than the theoretical value and may be correlated with the resolution. In order to be able to detect and analyse ice rings, which only occur within certain resolution ranges (see Table 1 ▸), it is useful to ‘remove’ the effect of such behaviour. To perform this, the local average standardized mean is estimated as a function of resolution and compared with the observed standardized mean in a given bin. In the current implementation, this resolution-dependent function *f* is calculated by performing interquartile-range filtering and robust Gaussian smoothing on the standardized mean after excluding the potential ice-ring ranges. Interpolation then allows estimates of the standardized mean to be achieved for each bin located within the potential ice-ring ranges.

For each bin, an ice-detection score is then calculated, *S* = *N* ^1/2^(〈*I*_obs_〉*s* ^−1^ − *f*), where the factor *N* ^1/2^ accounts for differences in the number of observations per bin, thus stabilizing the score across all resolution ranges. This score essentially measures the departure from the typical shape of the intensity distribution in the given data set, and can be loosely interpreted as a *Z*-score. This is sensitive to resolution ranges that exhibit sudden sharp changes in the intensity distribution, thus facilitating the detection and assessment of ice rings.

**Figure 8:**
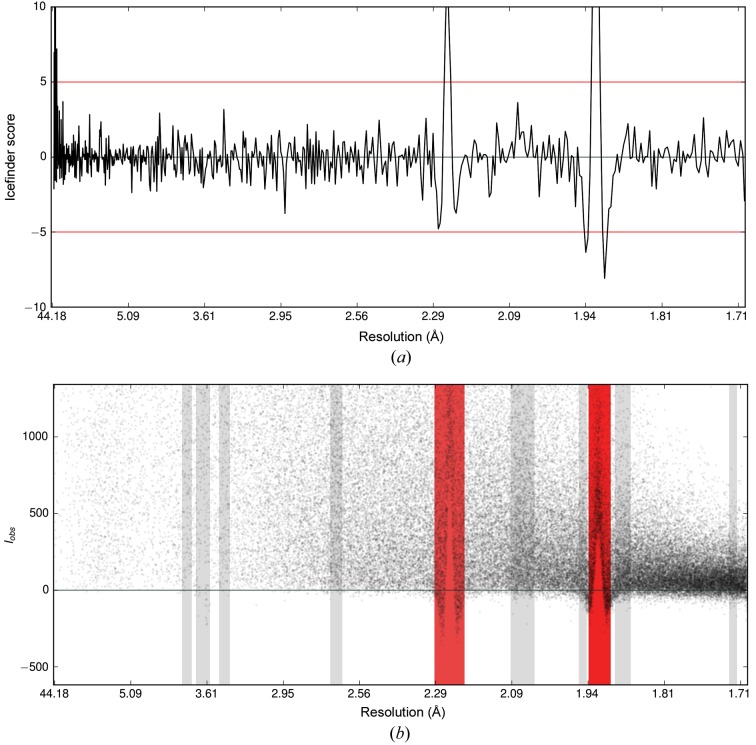
Automatic detection of ice rings in *AUSPEX* for PDB entry 3jqy. (*a*) Plot of the ice-detection score [‘Icefinder score’, black line;* S* = *N* ^1/2^(〈*I*_obs_〉*s* ^−1^ − *f*)] against resolution. The threshold of ±5 is marked by horizontal red lines. (*b*) Plot of *I*_obs_ against resolution with potential ice-ring resolution ranges in grey and flagged resolution ranges in red. The ice rings are clearly visible in both plots.

Owing to poor background estimation, the presence of ice rings can cause an increase or decrease in the mean intensity 〈*I*_obs_〉 (see Fig. 3 ▸) but not in the standard deviation of the intensity distribution. Consequently, the standardized mean and therefore *S* should increase or decrease in the presence of ice rings. However, the standard deviation can be increased or decreased relative to the mean by other problems in data processing, resulting in a particularly low score. Consequently, both positive and negative extreme score outliers are identified and flagged red in the plots (the default outlier threshold is ±5), as shown in Fig. 5 ▸, which shows a typical output. In Fig. 8 ▸, the ice-detection score *S* = *N* ^1/2^(〈*I*_obs_〉*s* ^−1^ − *f*) is shown together with the plot of *I*_obs_ against resolution for PDB entry 3jqy.

### 5.2. Comparison to other programs   

We selected another test set of 200 random data sets from the PDB for which intensity values had been deposited (test set C; see Supporting Information). By visual inspection of the *AUSPEX* plots, 45 of these contained ice rings, some of which were very weak. This was a similar fraction as found previously by visual inspection of test set B.

Of these 45 structures, six had missing data owing to the omission of entire resolution shells from the data.

Each of the 200 structures was analysed with *AUSPEX*, *CTRUNCATE* and *phenix.xtriage*; the results are shown in Table 2 ▸. *CTRUNCATE* gives a large number of false positives; *phenix.xtriage* applies more rigid criteria, resulting in fewer false positives but also more false negatives. *AUSPEX* performs more consistently in the four categories of false/correct positives and false/correct negatives. The *AUSPEX* implementation of automatic ice-ring detection is still inferior to the visual inspection of *AUSPEX* plots.

### 5.3. Testing of the algorithm against a large number of data sets   

Using this method, a large part of the PDB was evaluated. We found that 19% (5438 out of 28 895) of data sets with intensities deposited were suspected to have contamination owing to ice. This percentage is in keeping with the results from our more limited visual inspection of intensity *versus* resolution plots. This is a significant fraction which remains relatively consistent even in recent depositions in the PDB, demonstrating that this pathology is generally overlooked, presumably owing to a lack of necessary diagnostics, and that more sophisticated background-determination algorithms are needed to improve intensity estimation.

### 5.4. Limitations   

*AUSPEX* identifies all resolution ranges where ice rings are typically observed for hexagonal or cubic ice (see Table 1 ▸) with an associated score *S* outside a given threshold range (the default is ±5).

*AUSPEX* will not identify Debye–Scherrer rings from sources other than ice. Such rings can be caused, for example, by the crystallization plates used in *in situ* screening or by sample holders. Since *AUSPEX* only searches for ice rings in the expected resolution ranges (Table 1 ▸), it also cannot automatically detect ice rings if the wavelength or detector distance employed in data processing are wrong. However, *AUSPEX* could be extended in future to allow the detection of other phenomena such as rings caused by detergents and lipids, as used for example in membrane-protein crystallization.

If there is any doubt over the presence of an ice ring, the plot of the intensity distribution against resolution output by *AUSPEX* should be examined (see above).

## 6. Conclusion   

Even after more than 20 years of specific research to minimize the influence of ice diffraction in macromolecular crystallo­graphy (Mitchell & Garman, 1994 ▸), ice-ring artefacts were present in roughly 20% of 400 data sets (test sets B and C) chosen randomly from the PDB (as found by visual inspection of plots of *I*_obs_ *versus* resolution). A similar percentage (19%) was obtained when 28 895 data sets from the PDB for which intensities had been deposited were evaluated with the automatic ice-ring detection implemented in *AUSPEX*.

Optimization of cryoconditions so as to avoid ice rings is hampered by the difficulty in recognizing their presence on diffraction images, in particular images from modern pixel-array detectors, or from scaling statistics. In order to address this problem, a new software tool, *AUSPEX*, has been developed to facilitate ice-ring detection, allowing visual inspection of the intensity (or amplitude) distribution *versus* resolution as well as automatic ice-ring detection. The automatic ice-ring detection is arguably an improvement over current methods, although visual inspection of *AUSPEX* plots is presently the most reliable detection method.

The program can be used after scaling to check for data pathology, helping the user to decide whether it is necessary to re-integrate and rescale. It is also useful when looking at data sets that have already been solved in order to check the quality of the data underlying a model.

*AUSPEX* can also be used to investigate the structure and the distribution of errors within crystallographic data sets. The examples given illustrate effects associated with the multiplicity of measurements as well as the conversion from intensities to amplitudes. Although there is little direct evidence to suggest that these effects have a negative influence on structure solution using current software programs, there is clearly scope to improve the estimation of measurement errors in diffraction data (Waterman & Evans, 2010 ▸).

Furthermore, *AUSPEX* has inspired the development of an improved approach to background estimation that has been implemented in *DIALS* (Parkhurst *et al.*, 2017 ▸).

The program will be released in the near future as part of *CCP*4 (Winn *et al.*, 2011 ▸).

## Supplementary Material

Description of test sets.. DOI: 10.1107/S205979831700969X/hi5647sup1.pdf

## Figures and Tables

**Table 1:** Resolutions used in *AUSPEX* ice-ring identification; each bin has a start and end value, which have been manually curated

Bin No.	Start (Å)	End (Å)
1	3.953	3.807
2	3.753	3.581
3	3.482	3.371
4	2.692	2.635
5	2.294	2.209
6	2.094	2.041
7	1.954	1.939
8	1.935	1.897
9	1.890	1.863
10	1.723	1.712
11	1.527	1.516
12	1.476	1.465
13	1.446	1.434
14	1.372	1.365
15	1.305	1.292
16	1.285	1.247
17	1.240	1.217
18	1.186	1.162
19	1.136	1.119
20	1.099	1.067
21	1.052	1.029
22	1.017	1.011
23	1.000	0.984
24	0.981	0.975
25	0.973	0.966

**Table 2:** Results of automatic ice-ring detection in *phenix.xtriage* (Zwart *et al.*, 2005 ▸) and *CTRUNCATE* (Winn *et al.*, 2011 ▸) for 200 cases randomly selected from the PDB, as described in §[Sec sec5.2]5.2 There were 42 data sets that contained ice rings (see test set C in the Supporting Information). Six cases where data at ice-ring resolutions had been omitted were included in this test set. *phenix.xtriage* gives a message if only one ice ring has been found instead of an ice-ring warning; for the purposes of this test these messages were treated as ‘positives’.

	Correct positives	Correct negatives	False positives	False negatives
*phenix.xtriage*	14/42	145/158	13	28
*CTRUNCATE*	23/42	88/158	70	19
*AUSPEX*	25/42	143/158	14	17

## Footnotes

1 Auspex. Latin: diviner; augur; a person who observes birds in order to foretell the future.
