# Supplementary material for: AUSPEX: a graphical tool for X-ray diffraction data analysis
Source: Acta Crystallogr D Struct Biol. 2017 Aug 8;73(Pt 9):729–37. doi: 10.1107/S205979831700969X (PMC5586246; doi:10.1107/S205979831700969X)
Supplement: Supplementary file 1 [file d-73-00729-sup1.pdf]

STRUCTURAL BIOLOGY

Volume 73 (2017)

Supporting information for article:

AUSPEX: a graphical tool for X-ray diffraction data analysis

Andrea Thorn, James Parkhurst, Paul Emsley, Robert A. Nicholls, Melanie Vollmar, Gwyndaf Evans and Garib N. Murshudov

Test set A – Preliminary Study

156 data sets from the JCSG, collected on pixel detectors; evaluated were plots of F_{obs} vs. resolution. “Data were omitted at ice ring resolutions” refers to the deposited data set in the PDB missing resolution shells of X-ray data.

**PDB**	**remarks**
3R4K	
3RH7	
3SNK	
4DAD	
4DGU	
4DJN	
4DN6	ice rings visible
4DWE	
4E0E	
4E2E	ice rings visible
4E5V	
4E6E	
4E6F	
4E72	
4E9K	
4EBG	
4ECF	
4ECN	
4ECO	
4EF1	ice rings visible
4EF2	
4EI0	
4EPS	
4EPZ	ice rings visible
4EXR	
4EZG	ice rings visible
4F53	
4FD0	
4FDW	
4FDY	
4FJ6	

4FMR	ice rings visible
4FMZ	
4FSS	
4FTD	
4G2A	
4G5A	
4GBS	
4GHB	
4GHN	
4GL3	
4GOQ	
4GOT	ice rings visible
4GT6	
4H08	
4H1X	ice rings visible
4H3W	ice rings visible
4H40	
4H4J	
4H87	
4HBS	
4HF7	ice rings visible
4HPE	
4HXC	ice rings visible
4HYZ	
4I95	
4IEJ	ice rings visible
4JHY	
4JM1	
4JON	
4JPQ	
4JQS	
4JRL	
4JX0	
4JX2	
4K3F	

4K4K	
4K61	
4KH8	
4KH9	
4KQ7	
4KQT	
4KW2	ice rings visible
4KWY	
4L1N	
4L3R	
4L8J	
4L8N	
4LB8	
4LBA	
4LER	
4LG3	
4LGQ	
4LII	
4LJX	
4LQX	
4LQZ	
4LR4	
4M0H	
4M8K	
4M8R	
4MDW	
4ME9	
4MJF	
4MJG	data omitted at ice ring resolutions
4MRU	
4MU9	
4MUD	
4MXN	
4MXT	
4MYR	

4N0P	
4N0R	
4NKP	
4NRD	
4NW4	
4O2T	
4OBI	
4OBM	
4OLE	
4ONZ	
4OO3	
4OPM	ice rings visible
4OPW	
4ORL	
4OTE	
4OUQ	
4POI	ice rings visible
4PQX	
4PS6	ice rings visible
4PUC	data omitted at ice ring resolutions
4PUX	
4PW1	
4PXY	
4Q0Y	
4Q1Z	
4Q20	
4Q34	
4Q53	
4Q98	
4Q9A	
4QAN	
4QDY	
4QEY	
4QFU	
4QHZ	

4QJR	
4QOA	
4QPV	
4QRJ	
4QRL	
4QU6	
4QU7	
4QVU	
4R0K	
4R1K	
4R4G	
4R5O	
4R7F	
4R8O	
4RAA	
4RBO	
4RWV	
4YGT	
4YGU	
4YOD	

Test set B – Frequency of Ice Rings

200 data sets for which intensities were deposited, selected at random from the PDB. Visually inspected valuated were plots of I_{obs} vs. resolution. "Data were omitted" refers to the deposited data set in the PDB missing resolution shells of X-ray data.

PDB	remarks	collection year
425D		1995
1O1I		1998
1EBV		1999
1R5G		1999
1HJB		2000
3ZOW		2001
4DP0		2002
1SDV	data were omitted; additional ice rings visible	2002
1NRJ	ice rings visible and shifted from the usual resolution range	2002
3FEI		2002
1OO0		2002
3G8I		2002
1UO0		2002
2FVD		2002
4LA0		2003
1PW4		2003
1OIY		2003
1SCZ		2003
1V98		2003
1YLT		2004
3DKX		2004
4GLD		2004
1YKZ		2004
2ATP		2004
2W1Y		2004
2EHW		2004
2AFU	ice rings visible	2004
2NUE		2005
2ATK		2005
2EG3		2005
3OQ1		2005
2HRH		2005
2BDQ	ice rings visible	2005
2IEW	ice rings visible	2005
2IZP	data were omitted; additional ice rings visible	2005
2OUQ		2006
2I1Y	ice rings visible	2006
2QES		2006
2DW7		2006
2EJ1		2006
2I5A		2006

3GJW	ice rings visible	2006
2O1E		2006
2EIU		2006
3CI9	data were omitted	2006
2PT5	ice rings visible	2006
3ECN		2007
3B2R	ice rings visible	2007
2PP3		2007
2Z5L		2007
3NOQ		2007
3EE6		2007
3ZW3		2007
4GPG		2007
3FID	ice rings visible	2007
3BF8	ice rings visible	2007
3VFE		2007
2RAQ		2007
2R1V	data were omitted; additional ice rings visible	2007
4QXM		2007
2WY1		2007
2Y33		2008
3VT8		2008
3LY7	ice rings visible	2008
3GHE		2008
3QWX	ice rings visible	2008
3KLJ		2008
2WIW		2008
3GGF		2009
3N2O	ice rings visible	2009
4DQG		2009
3TUA		2009
3U2H		2009
3W6I		2009
2XBJ		2009
3QME		2009
4I9U		2009
3ARF		2009
4G94		2009
4Z8S		2009
4G4Z		2009
4U10		2009
3AJH		2009
3MTL	ice rings visible and shifted from the usual resolution range	2009
3OYZ	ice rings visible	2009
3P12		2010
3TN0		2010
2XWJ		2010
4LCG	ice rings visible	2010

4BJZ		2010
4DGC		2010
5IVI	ice rings visible	2010
2XWR		2010
3V6Y	ice rings visible	2010
4QPD	ice rings visible	2010
4ED3		2010
4ZMK		2010
3TRU		2010
5L5N		2010
2YID		2010
2Y6R	ice rings visible	2010
4H9A		2011
4BQU		2011
5KAT		2011
3U3B		2011
4U2L		2011
5KJE		2011
4DZG		2011
3T6O		2011
4P59		2011
3VY5		2011
4RQ9		2011
5LGW	ice rings visible	2011
4WNV	ice rings visible	2011
4F3Q	ice rings visible	2011
4KNJ		2011
4H1E		2011
4REP		2011
4JH3		2011
4G81		2012
4UC9		2012
4WP5		2012
4HKA		2012
4JFT		2012
3WN2	ice rings visible and shifted from the usual resolution range	2012
4MZE		2012
4HG2		2012
3WSZ		2012
4IU4		2012
3WBN		2012
4Y7N	ice rings visible	2012
4KVR		2012
3ZIY		2012
4RRZ		2012
4UWQ		2012
3WBB		2012
4OCF	ice rings visible	2012

4PRH		2012
4KA8	ice rings visible	2012
4CXO		2012
4IP1		2012
4IO7		2012
4L2Z		2012
4P12		2012
4PJO		2013
4JD7	ice rings visible	2013
5EPN		2013
4M9E		2013
4KDS	ice rings visible	2013
4CVZ		2013
4CVZ		2013
4C9N	ice rings visible	2013
5CR0		2013
4N6P		2013
4QQL	ice rings visible	2013
4NEQ		2013
5CPK		2013
4NJ4		2013
4NS1		2013
4RLT		2013
4R1B	ice rings visible	2013
4XA1		2013
4CVO		2014
4P3V		2014
4Q8S		2014
5BQB		2014
5BND	ice rings visible	2014
4ZHG		2014
5AWF		2014
4RZH		2014
4Z90		2014
5CL0		2014
4X62	ice rings visible	2014
4WN2		2014
5ABN		2014
5DH6		2014
4XH6		2014
5EK4	ice rings visible and shifted from the usual resolution range	2014
4Y88		2014
5IW8		2015
5I3Y	ice rings visible	2015
5F13		2015
4HS2		2011
1RQN		N/A
2CJR		N/A

2PHF		N/A
2XJJ		N/A
3EUZ		N/A
3ZP8		N/A
4BWG		N/A
4CVL		N/A
4DO1	ice rings visible	N/A
4E85		N/A
4GR2		N/A
4QTF	ice rings visible	N/A
4UWS		N/A
5A30	ice rings visible and shifted from the usual resolution range	N/A
5ACV	ice rings visible	N/A
4IOM	ice rings visible	N/A
4OW9		N/A

Test set C – Automatic Detection of Ice Rings

200 data sets for which intensities were deposited, selected at random from the PDB. Visually inspected were plots of I_{obs} vs. resolution. “Data omitted” refers to the deposited data set in the PDB missing resolution shells of X-ray data.

PDB	Visual	phenix.xtriage	Ctruncate	Auspex	collection year	remarks
1RDR	negative	negative	negative	negative	1995	
1C6H	negative	positive	positive	negative	1995	
1DCR	negative	negative	positive	positive	1996	
1EQM	negative	negative	negative	negative	1998	
1R0Q	negative	negative	positive	negative	1998	
1O1P	negative	negative	negative	negative	1999	
1JG1	negative	negative	positive	negative	2000	
1SJB	negative	negative	negative	positive	2001	
1VAT	negative	negative	positive	negative	2001	
1K34	negative	negative	positive	negative	2001	
2XYB	negative	negative	negative	negative	2001	
2GN9	negative	negative	negative	negative	2002	
1N6L	negative	negative	positive	negative	2002	
1YM0	positive	negative	negative	negative	2002	
2AVQ	negative	negative	positive	negative	2002	
2BSM	negative	positive	negative	positive	2003	
2H9Y	negative	negative	negative	negative	2003	
1ZZO	negative	negative	positive	negative	2003	
2FBD	negative	positive	positive	negative	2003	
3N2U	positive	negative	positive	negative	2003	
1YI1	negative	negative	negative	negative	2003	
2FG4	negative	negative	positive	negative	2003	
1YUM	positive	negative	positive	positive	2004	
1YG9	negative	negative	negative	negative	2004	
1ZIQ	positive	negative	positive	positive	2004	
1W96	negative	negative	negative	negative	2004	
3FK3	negative	negative	positive	negative	2004	
1VQ8	positive	negative	negative	positive	2004	
2B15	negative	negative	positive	negative	2004	
2I2Z	negative	negative	negative	negative	2004	
1XEA	positive	positive	negative	negative	2004	
2WKN	negative	negative	positive	negative	2004	
1Z6L	positive	negative	positive	negative	2004	
2W5P	negative	negative	positive	negative	2004	
2FI4	negative	negative	negative	negative	2005	
2ZU2	negative	negative	negative	negative	2005	
2O6M	negative	negative	negative	negative	2005	
3BT0	negative	negative	positive	negative	2005	
2QEX	negative	negative	negative	negative	2005	
2HD6	negative	negative	negative	negative	2005	
2NPZ	negative	negative	negative	positive	2005	

2D5N	negative	negative	negative	negative	2005	
3P88	negative	negative	positive	negative	2005	
2EW0	negative	negative	positive	negative	2005	
2GP9	negative	negative	positive	negative	2005	
2GB9	negative	negative	positive	negative	2005	
2RCU	negative	positive	positive	negative	2005	data omitted
3VHR	negative	negative	negative	negative	2005	
2PI5	negative	negative	negative	negative	2005	
2Z6A	negative	negative	negative	negative	2005	
3I9U	negative	positive	positive	negative	2006	
2G6Q	negative	negative	positive	negative	2006	
3BBM	negative	negative	negative	negative	2006	
3VHC	negative	negative	positive	negative	2006	
4KRD	negative	negative	positive	negative	2006	
3EHB	positive	negative	positive	positive	2006	
4PMN	negative	negative	negative	negative	2006	
2PNX	negative	negative	positive	negative	2007	
2VW9	positive	positive	positive	negative	2007	data omitted
5FDQ	negative	positive	negative	negative	2007	
3DRE	negative	negative	positive	negative	2007	
2ZKS	positive	negative	negative	negative	2008	ice rings shifted
3IFU	negative	negative	negative	negative	2008	ring outside ice ring resolutions
3A65	negative	negative	negative	negative	2008	
3DET	negative	negative	negative	negative	2008	
3TOG	negative	negative	positive	positive	2008	
3GL9	negative	positive	positive	negative	2008	
2XTE	negative	negative	negative	negative	2008	
4EX6	negative	negative	positive	negative	2008	
3M9S	negative	negative	negative	negative	2008	
3N04	negative	negative	positive	negative	2008	
4DW4	positive	positive	positive	positive	2008	
5D1P	negative	negative	negative	negative	2009	
3RL5	negative	negative	positive	negative	2009	
3HGY	negative	positive	negative	negative	2009	
2X13	negative	negative	negative	negative	2009	
5CDT	negative	negative	negative	negative	2009	
3HNM	positive	negative	negative	negative	2009	
4NT2	negative	negative	positive	negative	2009	
3JQY	positive	positive	positive	positive	2009	
3RMO	negative	negative	positive	positive	2009	
5FN8	negative	negative	negative	negative	2009	
3T8V	negative	negative	negative	negative	2009	
3NCG	positive	positive	positive	positive	2009	data omitted
3NHW	negative	negative	negative	negative	2009	
3MNL	negative	negative	negative	negative	2009	
4F27	positive	positive	positive	positive	2009	
4L17	negative	negative	negative	negative	2009	

4EA7	negative	negative	positive	positive	2009	
3B1J	negative	negative	negative	negative	2009	
3LZ0	positive	negative	negative	positive	2009	
4AC2	positive	negative	positive	negative	2009	
3X42	negative	negative	positive	negative	2010	
4NZG	positive	positive	positive	positive	2010	
3MYT	positive	positive	positive	positive	2010	data omitted
3OSZ	negative	negative	negative	negative	2010	
3MT0	negative	negative	positive	negative	2010	
2XWA	negative	negative	negative	negative	2010	
4HA4	negative	negative	positive	negative	2010	
3Q3O	negative	positive	positive	negative	2010	
3NVS	positive	negative	negative	positive	2010	
4H5B	negative	negative	positive	negative	2010	
3O2N	negative	negative	positive	negative	2010	
4A14	negative	positive	positive	negative	2010	
5KC5	negative	negative	negative	negative	2010	
4F5J	negative	negative	negative	negative	2010	
4GFA	negative	negative	negative	negative	2010	
3PO9	negative	negative	negative	negative	2010	
4ELP	negative	negative	negative	negative	2010	
3TAL	negative	negative	negative	negative	2010	
3ZYM	positive	negative	negative	negative	2010	
3US1	negative	negative	negative	negative	2010	
4NXN	negative	negative	negative	positive	2010	
3SBI	negative	negative	positive	negative	2010	
3SYA	negative	negative	negative	negative	2010	
4GBA	positive	negative	negative	positive	2011	
3R3M	negative	negative	negative	negative	2011	
4E2W	negative	negative	positive	positive	2011	
3RIB	negative	negative	negative	negative	2011	
4BI2	negative	negative	negative	negative	2011	
3SV9	negative	negative	negative	negative	2011	
3SUF	negative	negative	negative	negative	2011	
4MCF	negative	negative	negative	negative	2011	
4MW7	negative	negative	negative	negative	2011	
4JC7	negative	negative	negative	negative	2011	
4MIY	negative	negative	negative	negative	2011	
4X7K	negative	negative	negative	negative	2011	
4ES4	negative	negative	negative	negative	2011	
4K37	positive	negative	negative	negative	2011	
3U12	negative	negative	positive	negative	2011	
4AFD	negative	negative	positive	negative	2011	
4M1O	negative	negative	negative	negative	2011	
4TVQ	positive	negative	negative	negative	2011	
4MB7	negative	negative	negative	negative	2011	
5C1X	negative	negative	negative	negative	2012	
4LO3	negative	positive	negative	negative	2012	

4HKX	negative	negative	positive	positive	2012	
3WE4	negative	negative	negative	negative	2012	
4WIE	positive	positive	negative	negative	2012	
4IAZ	negative	negative	negative	negative	2012	
3WF7	negative	negative	negative	negative	2012	
4KGR	negative	negative	negative	positive	2012	
4ISZ	negative	negative	negative	negative	2012	
4IPG	negative	negative	positive	negative	2012	
4LA6	negative	negative	negative	negative	2012	
4LY8	positive	negative	negative	positive	2012	
4J7Y	positive	positive	negative	positive	2012	
4IKM	negative	negative	positive	negative	2012	
4HJX	negative	negative	negative	negative	2012	
3W7V	negative	negative	positive	positive	2012	
4Q75	positive	negative	positive	negative	2012	
4MVS	negative	negative	negative	negative	2012	
4J29	negative	negative	positive	negative	2012	
4X6B	positive	negative	negative	positive	2012	
4OU1	positive	negative	positive	positive	2013	
4L55	negative	negative	negative	negative	2013	
4NZ0	negative	negative	positive	negative	2013	
4CCM	positive	negative	negative	negative	2013	
4OQ4	negative	negative	positive	negative	2013	
4YCU	positive	negative	negative	positive	2013	
4M00	positive	positive	positive	positive	2013	
4Q3O	positive	negative	negative	positive	2013	
4XET	negative	negative	positive	negative	2013	
4P66	negative	negative	negative	negative	2013	
4X8W	negative	positive	positive	negative	2013	
4NSV	negative	negative	positive	negative	2013	
4MH4	negative	negative	positive	negative	2013	
4YYA	negative	negative	negative	negative	2013	
4WV8	negative	negative	negative	negative	2013	
4YW6	positive	negative	positive	positive	2013	data omitted
4O75	negative	negative	positive	negative	2013	
4QYM	negative	negative	positive	negative	2013	
4CRH	negative	negative	positive	negative	2014	
4XF9	negative	negative	positive	negative	2014	
5A7S	negative	negative	positive	positive	2014	
5FJS	negative	negative	negative	negative	2014	
5CES	negative	negative	positive	negative	2014	
5HF6	negative	negative	negative	negative	2014	
4RYL	positive	positive	positive	positive	2014	
5D0C	positive	positive	positive	negative	2014	data omitted
4ZVN	negative	negative	negative	negative	2014	
5ELF	negative	negative	negative	negative	2014	
4Z3M	negative	negative	negative	negative	2014	
5C8X	negative	negative	positive	negative	2015	

5E5T	negative	negative	positive	negative	2015	
5FAQ	positive	negative	positive	positive	2015	
1I0V	negative	negative	positive	negative	N/A	
2XDS	negative	negative	positive	negative	N/A	
3AP3	negative	negative	negative	negative	N/A	
3FD5	negative	positive	positive	negative	N/A	
3IKT	positive	negative	negative	positive	N/A	
3ZXH	positive	negative	positive	positive	N/A	
3ZZS	negative	negative	positive	positive	N/A	
4AW9	positive	positive	positive	negative	N/A	data omitted
4AWA	negative	negative	negative	negative	N/A	
4G1N	negative	negative	negative	negative	N/A	
4KWC	negative	negative	positive	negative	N/A	
4N9R	positive	negative	positive	negative	N/A	
4OQ5	negative	negative	negative	negative	N/A	
4UT0	negative	negative	negative	negative	N/A	
